# Editorial: Transcatheter Aortic Valve Implantation—Current Challenges and Future Directions

**DOI:** 10.3389/fcvm.2021.748376

**Published:** 2021-09-07

**Authors:** Richard J. Jabbour, Azeem Latib, Antonio Colombo, Vasileios Panoulas

**Affiliations:** ^1^Cardiology Department, Imperial College Healthcare Trust, London, United Kingdom; ^2^Montefiore Medical Center—Albert Einstein College of Medicine, New York, NY, United States; ^3^Department of Biomedical Sciences, Humanitas University, Milan, Italy; ^4^Humanitas Clinical and Research Center Istituto Clinico Humanitas Humanitas Cancer Center, Milan, Italy; ^5^Guys and St Thomas' NHS Foundation Trust, Royal Brompton and Harefield Hospitals, Harefield, United Kingdom

**Keywords:** TAVI, TAVR, aortic stenosis, valve, transcatheter

Transcatheter aortic valve implantation (TAVI) has rapidly evolved to become the treatment of choice for patients with severe aortic stenosis (1). Despite this, TAVI with current generation devices is unlikely to become suitable for all patients, including those with hostile access sites and unfavorable aortic root anatomies, concomitant mitral or tricuspid valve disease requiring repair or replacement, or associated aortopathy requiring aortic root surgery. In addition, the inclusion of younger lower-risk patients into the realm of TAVI brings its own challenges, including treatment of true bicuspid aortic valve disease, future coronary access, durability and risks associated with long term permanent pacing. Accordingly, we decided to create this Research Topic to try to illustrate some of the current challenges and future directions of the therapy. Seven articles of timely importance are included.

Firstly, 2 articles describe the rapid expansion of TAVI in the United States and the efficacy of TAVI vs. surgical aortic valve replacement (SAVR) in low to intermediate surgical risk patients.

In the first article, Elbaz-Greener et al. describe trends in the utilization of SAVR and TAVI over time in the United States. They illustrate how TAVI use has expanded rapidly and overtook SAVR in 2017 to become the dominant treatment choice for severe AS. Utilizing data from the largest all-payer inpatient database in the United States, they identified a weighted total of 542,734 patients who underwent SAVR. Their data shows a relatively steady trend in utilization of SAVR in AS patients during the early TAVI era (2011–2014) with a significant downward trend in the following years (2015–2017). In contrast, TAVI use increased steadily over time. In the latter period there was an increased prevalence of comorbidities in the TAVI group; however, complications and mortality rate significantly decreased over time. This is likely related to increasing operator familiarity and newer generation devices that have better profiles and outcomes. Based on the data provided in this study TAVI will continue to expand and the gap between TAVI and SAVR diverge further.

The second article is a meta-analysis where Lou et al. describe the safety and efficacy of transcatheter vs. surgical aortic valve replacement in low to intermediate surgical risk patients with primary outcome of mortality. For patients deemed at low surgical risk, TAVI was associated with a lower mortality rate at 1 year. For patients with an intermediate surgical risk, mortality rates were equivalent between groups. TAVI was associated with decreased rates of bleeding and renal failure but increased rates of reintervention and major vascular complications. Regarding myocardial infarction and stroke, rates were similar between groups. Whilst it is important to show equivalence in low-risk groups, long term data are eagerly awaited. Whilst randomized data to 8 years exists, longer-term data from multiple randomized trials in low to intermediate risk patients will be the key to lowering the age limits in recommending TAVI over SAVR (2).

The other articles in the collection describe the current challenges of TAVI and periprocedural considerations including performing PCI and antithrombotic regimens post TAVI.

Prosthesis-patient mismatch (PPM) develops when the effective orifice area of the prosthetic valve is relatively small in relation to body size. This results in the generation of higher-than-expected gradients through normally functioning prosthetic valves and has been associated with worse outcomes (3). PPM has been known to affect both SAVR and TAVI and accordingly, Leone et al. provide a detailed review on the patient prosthesis mismatch post-TAVI. They describe multimodality assessment, epidemiology and risk factors, and describe various ways to mitigate the risk including patient selection, pre-procedural planning, valve choice and sizing. Related to this review, Ruge et al. describe their single centre outcomes of bioprosthetic valve fracturing in a valve-in-valve cohort of 67 consecutive patients with valve fracturing attempted in 15 cases. Valve fracturing was successful in 53.3%, indicating some of the challenges of performing this procedure in the real world, especially in failed Perimount (Carpentier-Edwards) valves. Despite a reduction in valve gradients when compared to standard postdilatation, long term data are still eagerly awaited to see if it translates into improved hard endpoint outcomes.

The second meta-analysis in this collection by Zhang et al., provide a timely update on the safety and efficacy of a dual vs. single antiplatelet strategy post-TAVI. Twelve studies of 20,766 patients were included in their meta-analysis. Compared with single antiplatelet therapy (SAPT), post-TAVI dual antiplatelet therapy (DAPT) was associated with increased risks of major or life-threatening bleeding without additional benefits of reducing thrombotic events (4). Based on emerging data, guidelines are starting to recommend SAPT over DAPT in patients without recent PCI, and it has indeed been current practice in many centres to prescribe SAPT post TAVI for some time.

In the 6^th^ article, Li et al. describe anatomical predictors of valve malpositioning of the self-expanding Venus A-Valve. A conical left ventricular outflow tract and tall aortic sinuses were strong anatomical predictors of malpositioning during self-expandable TAVR with the presence of both predictors associated with a very high risk. This article highlights the emerging importance of valve and aortic root anatomy in deciding suitability for TAVI over SAVR.

Finally, El-Medany et al., report an interesting case of complex PCI post TAVI using intravascular imaging. They then describe the procedural and device factors and anatomical factors associated with ease of coronary access and considerations and approach for PCI post-TAVI. With appropriate training PCI can be performed relatively easily in patients with prior TAVI. Downsizing catheters (e.g., JL3.5 instead of JL4) and more importantly the use of guide catheter extension technology are recommended (5).

The constellation of the above articles emphasize the growing importance and efficacy of TAVI and illustrate some of the challenges with current technology.

## Author Contributions

RJ and VP wrote the editorial. AL and AC critically revised it. All authors contributed to the article and approved the submitted version.

## Conflict of Interest

The authors declare that the research was conducted in the absence of any commercial or financial relationships that could be construed as a potential conflict of interest.

## Publisher's Note

All claims expressed in this article are solely those of the authors and do not necessarily represent those of their affiliated organizations, or those of the publisher, the editors and the reviewers. Any product that may be evaluated in this article, or claim that may be made by its manufacturer, is not guaranteed or endorsed by the publisher.
